# Daily and meal-based assessment of dairy and corresponding protein intake in Switzerland: results from the National Nutrition Survey menuCH

**DOI:** 10.1007/s00394-020-02399-7

**Published:** 2020-10-08

**Authors:** Dilara Inanir, Ivo Kaelin, Giulia Pestoni, David Faeh, Nadina Mueller, Sabine Rohrmann, Janice Sych

**Affiliations:** 1 Institute of Food and Beverage Innovation, ZHAW School of Life Sciences and Facility Management, Einsiedlerstrasse 34, 8820 Waedenswil, Switzerland; 2 Institute of Applied Simulation, ZHAW School of Life Sciences and Facility Management, Schloss 1, 8820 Waedenswil, Switzerland; 3 Division of Chronic Disease Epidemiology, Epidemiology, Biostatistics and Prevention Institute, University of Zurich, Hirschengraben 84, 8001 Zurich, Switzerland; 4 Health Department-Nutrition and Dietetics, Bern University of Applied Sciences, Bern, Switzerland

**Keywords:** Dairy, Dairy protein, Protein intake, menuCH

## Abstract

**Purpose:**

Dairy contributes to daily protein and provides important minerals and vitamins. Using data of the National Nutrition Survey in Switzerland (menuCH), we aimed to describe intakes of dairy and its subcategories, to compare daily and per-meal dairy protein with total protein intake, and to investigate associations between energy-standardized dairy intake and sociodemographic, lifestyle and anthropometric factors.

**Methods:**

From two 24-h dietary recalls, anthropometric measurements, and a lifestyle questionnaire from a representative sample (*n* = 2057, 18–75 years), we calculated daily and energy-standardized means and standard error of the means for dairy, its subcategories (milk, yoghurt and cheese), and compared daily and per-meal dairy protein with total protein intake. Associations were investigated between dairy intake (g/1000 kcal) and sociodemographic, lifestyle and anthropometric factors by multivariable linear regression.

**Results:**

Dairy intake provided 16.3 g/day protein with cheese contributing highest amounts (9.9 g/day). Dairy protein intake was highest at dinner (6.3 g/day) followed by breakfast, lunch and snacks (4.3, 3.3 and 2.4 g/day, respectively). Per meal, total protein reached the amounts suggested for improving protein synthesis only at dinner and lunch (33.1 and 28.3 g/day, respectively). Energy-standardized dairy intake was 20.7 g/1000 kcal higher for women than men (95% CI 13.2; 28.1), 24.3 g/1000 kcal lower in the French than German-speaking region (95% CI − 32.4; − 16.1), and also significantly associated with nationality, household type and smoking status.

**Conclusion:**

This first description of dairy consumption is an important basis for developing meal-specific recommendations, aimed to optimize dairy and protein intake especially for older adults.

**Electronic supplementary material:**

The online version of this article (10.1007/s00394-020-02399-7) contains supplementary material, which is available to authorized users.

## Introduction

Dairy products including milk, yoghurt and cheese are an important part of diet in Switzerland. They provide high-quality protein, vitamins (A, B12, riboflavin) and minerals (calcium, iodine, magnesium and potassium), at varying levels depending on product type. Dairy is generally considered to contribute positively to health [1] largely based on its nutritional properties. However, one major concern has been the high levels of saturated fats in many dairy products. Not all studies are consistent, but the current consensus is that dairy intake, including regular fat dairy products, is not associated with negative health outcomes, i.e., cardiovascular disease or type 2 diabetes, and in some cases, protective effects have been reported [2, 3]. These effects and other positive health outcomes linked to dairy consumption may differ between dairy subcategories. For example, there is particular interest in effects observed for consumption of fermented dairy products, such as yoghurt and cheese [4, 5].

Dairy proteins have a favorable amino acid profile for muscle synthesis [6, 7], especially important for the rapidly aging population who have higher protein requirements compared with younger adults [8]. Dietary protein recommendations are traditionally expressed on a daily basis, but interest towards protein intake per meal is increasing. Recent evidence supports that protein synthesis and possibly other metabolic benefits may be promoted by regular distribution of total protein across meals and with a suggested meal threshold of approximately 30 g [8–10]. However, only a few studies have examined dairy intake or its protein per meals [11, 12], and to our knowledge, no study has compared per-meal intakes of dairy protein with that of total protein.

In the first report of the Swiss National Nutrition Survey menuCH which summarized and compared consumption of all food groups with recommendations [13], mean dairy intake was two portions per day, which is one portion below the Swiss nutritional recommendation [13, 14]. Low adherence to dairy recommendations in Switzerland was reported earlier [15] and also in Europe and elsewhere [16–19]. Moreover, decreases in dairy consumption are expected in future due to the current trend towards plant-based diets [20].

The menuCH Survey is the first national, representative sample in Switzerland with dietary intake assessed by 24-HDR (24-h dietary recall) and with a detailed assessment of sociodemographic, lifestyle and anthropometric factors, therefore providing an opportunity for an in-depth study of dairy consumption. Given the high nutritional value of dairy and new perspectives of protein intake, this study aimed to describe intake of dairy and its subcategories using menuCH data, with a focus on their protein contributions compared with total protein per day and per meal. A second important aim was to investigate the associations between dairy intake and sociodemographic, lifestyle and anthropometric factors.

## Materials and methods

### Study design

This secondary analysis used data from the Swiss National Nutrition Survey menuCH, a cross-sectional population-based survey, carried out between January 2014 and February 2015, as described earlier [13]. A random sample of Swiss residents aged 18–75 years was recruited, representative of 35 strata (7 × 5): seven administrative regions of Switzerland (Lake Geneva, Midlands, Northwest, Zurich, Eastern, Central and Southern Switzerland) from three main language regions (German-, French- and Italian-speaking region) and five age groups: 18–29, 30–39, 40–49, 50–64 and 65–75 years old. From 13,606 individuals, 5496 were contacted by mail or phone, and the final study group was 2086 adults, corresponding to 38% participation rate [13]. Data from 2057 participants who completed the two 24-HDR were analyzed and reported following guidelines for Strengthening the Reporting of Observational Studies in Epidemiology-Nutritional Epidemiology (STROBE-nut) [62].

The survey protocol was approved by the ethics committee of the canton of Lausanne (Protocol 26/13) and by corresponding regional ethics committees (registered ISRCTN number 16778734) [61], and informed consent of all study participants was obtained.

### Dietary assessment

Survey methods included two non-consecutive 24-HDRs, performed by trained dietitians, conducted in person and two to six weeks later by telephone, distributed across seasons and weekdays. Participants also completed a questionnaire to assess dietary and lifestyle habits, and sociodemographic factors, and anthropometric measurements were taken [13]. A food picture book illustrating portion sizes and common household measures was used during the 24-HDR [21]. Supplement intake was not assessed, except for two questions in the questionnaire. The questionnaire assessed food avoidance, including reasons. Consumption of foods, recipes and ingredients was recorded using the software GloboDiet® (formerly EPIC-Soft®, version CH-2016.4.10, International Agency for Research on Cancer (IARC), Lyon, France) [22, 23], adapted for Switzerland (GloboDiet® trilingual databases dated 12.12.2016, IARC, Lyon, France; Federal Food Safety and Veterinary Office, Bern, Switzerland). Recipes were disaggregated into ingredients, according to standard recipes, and assigned to the corresponding food category defined in GloboDiet®. Each consumption was linked to the most appropriate food item in the Swiss Food Composition Database [24] to obtain intakes of energy, proteins, carbohydrates and fats. A pilot study was conducted to evaluate and optimize the methods of the survey [25] and data cleaning was done according to IARC recommendations [22].

### Definition of food categories

Our analysis focused on high-protein sources of the food group dairy, according to the Swiss food pyramid (fourth level) [14]. Three subcategories were analyzed: milk (milk, milk drinks and fermented milk drinks), yoghurt (yoghurt, sour milk products, cottage cheese and quark) and cheese (soft, semisoft and hard). This included dairy foods and dairy ingredients which were disaggregated from recipes (such as cheese from pizza) reported in the 24-HDR. Dairy-based desserts and cream were not considered in the analysis due to low amounts of protein. Due to their lower protein quality compared with animal sources, plant-based dairy alternatives were analyzed separately and named dairy alternatives.

### Definition of meals and snacks

During the 24-HDR, study participants self-reported the type of eating occasion as one of seven possibilities: before breakfast, breakfast, during the morning, lunch, during afternoon, dinner, during the evening or at night. Dairy, corresponding protein and total daily protein intakes were examined per meal and snacks by re-arranging the seven possible food intake occasions into four categories as follows: breakfast (before breakfast and breakfast), lunch, dinner, and snacks (morning, afternoon, evening or at night). Due to low amounts, dairy intakes at snacks were combined.

### Sociodemographic, lifestyle and anthropometric variables

Food consumption data were described with respect to sociodemographic, dietary and lifestyle habits based on results from the questionnaire, and anthropometric data. These data were also used to investigate associations between energy-standardized dairy intake and sociodemographic, lifestyle and anthropometric variables by multivariable regression analysis. The main variables were sex (men, women); three-language regions (German-, French- and Italian-speaking) determined by canton of residence (German-language: Aargau, Basel-Land, Basel-Stadt, Bern, Lucerne, St. Gallen, Zurich; French-language: Geneva, Jura, Neuchatel, Vaud; and Italian-language region: Ticino) and age determined by self-reported date of birth and analysed as four groups: 18–29, 30–44, 45–59, and 60–75 years. Following international standard protocols [13, 26], body weight and height were measured and used to calculate BMI (Body Mass Index), categorized as underweight (< 18.5 kg/m^2^), normal (18.5 kg/m^2^ ≤ BMI < 25.0 kg/m^2^), overweight (25.0 kg/m^2^ ≤ BMI < 30.0 kg/m^2^) and obese (≥ 30.0 kg/m^2^). Self-reported weight and/or height were used for pregnant (*n* = 14) or lactating women (*n* = 13) (values pre-pregnancy), or when measurements were not possible (*n* = 7). Other sociodemographic variables were nationality (Swiss, Swiss binational, non-Swiss), education (primary or no degree, secondary, tertiary), household type (living alone, couple without children, couple with children, one-parent family with children, adult living with parents, others, such as shared flat), gross household income (< 6000, 6000–13,000, > 13,000 Swiss francs/month). Lifestyle variables of interest were smoking status (never, former, current), currently on a weight-loss diet (yes, no) and self-reported health status, assessed as five levels but analyzed as two: very poor to medium and good to very good. Physical activity level was assessed by the International Physical Activity Questionnaire (short version, IPAQ) and categorized into low, moderate and high [27, 28].

### Data analysis

Using data from both interviews, mean and standard error of the mean (SEM) were used to describe the intake of total dairy, dairy subcategories, their respective protein contributions and total daily protein for the population and by sex, language region and age group per day and per meal. Dairy and protein intakes were standardized to g/1000 kcal to account for differences in energy intake. Daily protein intakes per kg body weight of participants were compared with recommendations of the German, Austrian and Swiss Nutrition Societies (DACH) [29], i.e.intake of ≥ 0.8 g protein per kg body weight for 19–64 years; and ≥ 1.0 g protein per kg body weight for ≥ 65 years of age.

A multivariable linear regression model was applied to investigate associations between energy-standardized dairy intake and selected socio-demographic, lifestyle and anthropometric variables, adjusted for sex, age group, language region, BMI category, nationality, education degree, household type, gross household income, physical activity level, smoking status, self-reported health status and diet status. To account for missing values for education degree (*n* = 3), civil status (*n* = 3), household type (*n* = 3), smoking status (*n* = 4), self-reported health status (*n* = 4), currently on a diet (*n* = 4), physical activity (*n* = 473) and gross household income (*n* = 585), multiple imputation by chained equations (*m* = 25) was performed [30].

All data were weighted for sex, age, marital status, major area of Switzerland, nationality and household size, and consumption data were additionally weighted for season and weekday. This corrected for sampling design and non-response, allowing a more accurate extrapolation of the results from 2057 participants to 4,627,878 individuals of the population [31].

All analyses were conducted using R-software (version 3.6.1), with additional R-packages for the weighting (*stats*) [32], histograms (*weights*) [33] and multiple imputation (*mice*) [30]. Normality of data distribution was checked by Shapiro–Wilk test [34].

## Results

Table 1 summarizes the sociodemographic, lifestyle and anthropometric characteristics of the study population which included 2057 participants who represented 4,627,878 individuals after weighting. The majority of the study sample was Swiss, from the German-speaking region, middle-aged, highly educated, non-smokers, had a normal BMI and self-reported a good-to-very good self-reported health status.

**Table 1:** Description of sociodemographic, lifestyle and anthropometric characteristics of the study participants (*n* and %)

	Crude	Weighted^a^
Number of participants with two 24-HDR (*n*)	2057	–
Number of people, weighted analysis (*n*)	–	4,627,878
Sex		
Men	45.4%	49.8%
Women	54.6%	50.2%
Language regions^b^		
German	65.2%	69.2%
French	24.4%	25.2%
Italian	10.4%	5.6%
Age groups (years)^c^		
18–29	19.4%	18.8%
30–44	25.9%	29.9%
45–59	30.4%	29.8%
60–75	24.3%	21.6%
BMI categories^d^		
Underweight (BMI < 18.5 kg/m^2^)	2.5%	2.4%
Normal (18.5 ≤ BMI < 25.0 kg/m^2^)	54.2%	54.1%
Overweight (25.0 ≤ BMI < 30.0 kg/m^2^)	30.6%	30.6%
Obese (BMI ≥ 30.0 kg/m^2^)	12.7%	12.9%
Nationality		
Swiss only	72.5%	61.4%
Swiss binational	14.4%	13.8%
Non-Swiss	13.0%	24.8%
Education, highest degree		
Primary or no degree	4.3%	4.7%
Secondary	47.1%	42.6%
Tertiary	48.5%	52.6%
Household type		
Living alone	16.1%	18.1%
Adult living with parents	7.8%	7.1%
Couple without children	33.6%	31.7%
Couple with children	33.1%	32.8%
One-parent family with children	4.6%	4.4%
Others^e^	5.4%	5.7%
Gross household income (CHF/month)		
< 6000	16.8%	17.7%
6000–13,000	40.9%	39.8%
> 13,000	13.9%	14.9%
No answer	28.4%	27.6%
Physical activity level		
Low	12.2%	15.1%
Moderate	22.1%	22.7%
High	40.2%	40.3%
No answer	25.5%	22.0%
Smoking status		
Never	44.4%	42.9%
Former	33.4%	33.6%
Current	21.9%	23.3%
Self-reported health status		
Very poor to medium	13.2%	12.7%
Good to very good	86.6%	87.1%
Currently on a diet		
Yes	5.5%	5.4%
No	94.3%	94.4%

*24-HDR* 24-h dietary recall, *BMI* body mass index, *CHF* Swiss Francs

^a^Percentages are weighted for sex, age, marital status, major area of Switzerland, household size, and nationality

^b^German language—cantons of Aargau, Basel-Land, Basel-Stadt, Bern, Lucerne, St. Gallen, and Zurich; French language—Geneva, Jura, Neuchatel, and Vaud; and Italian-language region –Ticino

^c^Self-reported age on the day of completion of the questionnaire

^d^BMI by measured height and weight, or self-reported when measurements were not possible or pre-pregnancy weight (lactating and pregnant women)

^e^Others represents study participants living without a partner and parents (e.g. in a shared flat)

Almost all participants (96.7%) reported dairy intake and this consumption was not normally distributed (Online Resource, S1 and S2). Table 2 shows mean intake of dairy was 216.5 g/day with highest intake of milk, followed by yoghurt and cheese. Energy-standardized amounts consumed of total dairy, its subcategories and corresponding protein showed considerable differences between sexes. Although daily intake of dairy (g/day) was higher in men than women, energy-standardized intake (g/1000 kcal) was higher for women. Comparing language regions, energy-standardized dairy intake was lowest in the French- and highest in the German-speaking region. Milk and yoghurt were highest in the German-speaking region, whereas cheese intake was highest in the Italian-speaking region (g/1000 kcal). Energy-standardized data suggest slightly higher milk intake in younger participants (18–29 years), but slightly higher yoghurt and cheese intake in older participants (45–59 and 60–75 years).

**Table 2:** Mean intake of dairy, its subcategories, and corresponding protein compared with total protein intake; and numbers of participants with protein intake below recommendations, by sex, language region and age group (g/1000 kcal, g/day, *n* and %)

		All (*n* = 2057)		Sex				Language regions^a^						Age groups (years)^b^							
				Men (*n* = 933)		Women (*n* = 1124)		German (*n* = 1341)		French (*n* = 502)		Italian (*n* = 214)		18–29 (*n* = 400)		30–44 (*n* = 533)		45–59 (*n* = 625)		60–75 (*n* = 499)	
		Mean	SEM	Mean	SEM	Mean	SEM	Mean	SEM	Mean	SEM	Mean	SEM	Mean	SEM	Mean	SEM	Mean	SEM	Mean	SEM
g/1000 kcal	Foods																				
	Dairy	99.7	1.8	88.5	2.6	110.8	2.5	106.0	2.4	83.0	3.2	98.5	5.4	100.5	4.1	96.9	3.7	99.3	3.1	103.4	3.5
	Milk^c^	51.7	1.5	45.9	2.2	57.5	2.0	57.5	1.9	35.8	2.3	53.6	4.5	58.2	3.3	54.9	3.2	47.5	2.4	47.4	2.8
	Yoghurt^d^	29.0	0.9	23.7	1.2	34.4	1.4	29.5	1.1	29.3	1.9	22.6	2.7	25.8	2.0	24.9	1.7	30.6	1.7	35.5	2.0
	Cheese^e^	18.9	0.4	18.9	0.6	18.9	0.6	19.0	0.5	17.9	0.8	22.3	1.4	16.5	1.0	17.1	0.7	21.2	0.8	20.5	0.8
	Dairy alt.^f^	3.7	0.4	2.3	0.5	5.0	0.7	2.9	0.5	5.8	1.1	3.5	1.1	2.8	0.7	4.2	0.9	4.7	1.0	2.2	0.6
	Protein																				
	Dairy	7.3	0.1	6.9	0.2	7.8	0.2	7.6	0.1	6.6	0.2	7.8	0.4	6.8	0.3	6.8	0.2	7.8	0.2	7.9	0.2
	Milk^c^	1.7	0.0	1.5	0.1	1.9	0.1	1.9	0.1	1.2	0.1	1.8	0.1	1.9	0.1	1.8	0.1	1.6	0.1	1.6	0.1
	Yoghurt^d^	1.3	0.0	1.0	0.1	1.6	0.1	1.4	0.1	1.3	0.1	0.9	0.1	1.3	0.1	1.2	0.1	1.4	0.1	1.6	0.1
	Cheese^e^	4.3	0.1	4.3	0.1	4.3	0.1	4.3	0.1	4.1	0.2	5.1	0.3	3.6	0.2	3.9	0.2	4.8	0.2	4.8	0.2
g/day	Foods																				
	Dairy	216.5	4.1	221.5	6.8	211.5	4.9	234.3	5.3	174.2	7.5	191.4	10.0	220.4	9.4	215.0	8.4	217.1	7.2	214.3	8.4
	Milk^c^	113.4	3.4	115.7	5.8	111.2	3.0	128.4	4.5	75.8	5.7	102.1	8.0	128.9	7.9	121.4	7.3	105.9	5.8	99.4	6.6
	Yoghurt^d^	59.7	1.8	56.5	2.7	63.0	2.4	61.5	2.2	58.8	3.6	42.0	5.2	51.2	3.7	53.8	3.6	63.1	3.2	70.8	3.7
	Cheese^e^	43.3	1.1	49.3	1.8	37.3	1.2	44.4	1.3	39.6	2.1	47.3	3.4	40.2	2.7	39.9	1.9	48.2	2.1	44.1	1.9
	Dairy alt.^f^	7.2	0.9	5.0	1.1	9.4	1.3	6.0	1.0	10.5	2.0	6.7	2.3	5.7	1.4	8.7	1.9	9.3	1.9	3.6	1.0
	Protein																				
	Dairy	16.3	0.3	17.5	0.5	15.1	0.4	17.1	0.4	14.2	0.6	16.0	0.9	15.6	0.7	15.5	0.5	17.5	0.6	16.6	0.6
	Milk^c^	3.7	0.1	3.8	0.2	3.6	0.1	4.2	0.1	2.5	0.2	3.4	0.3	4.3	0.3	3.9	0.2	3.5	0.2	3.3	0.2
	Yoghurt^d^	2.7	0.1	2.5	0.1	2.9	0.1	2.8	0.1	2.6	0.2	1.8	0.3	2.5	0.2	2.5	0.2	2.8	0.2	3.1	0.2
	Cheese^e^	9.9	0.2	11.3	0.4	8.6	0.3	10.1	0.3	9.2	0.5	10.8	0.8	8.8	0.6	9.2	0.5	11.2	0.5	10.3	0.5
	Total protein	84.6	0.7	97.7	1.2	71.6	0.8	84.6	0.9	84.7	1.4	84.4	2.5	89.9	2.1	87.9	1.5	84.2	1.2	75.9	1.2
	Below protein rec.^g^	*n*	%	*n*	%	*n*	%	*n*	%	*n*	%	*n*	%	*n*	%	*n*	%	*n*	%	*n*	%
		434.6	21.3	192.6	18.6	242.1	24.1	309.6	22.3	97.3	18.2	27.8	23.4	49.8	13.4	100.4	16.5	104.1	17.2	180.3	40.8

All results weighted for sex, age, marital status, major area of Switzerland, nationality and household size, season and weekday

*SEM* standard error of the mean, *Dairy alt.* dairy alternatives and *rec* recommendation

^a^German language - cantons of Aargau, Basel-Land, Basel-Stadt, Bern, Lucerne, St. Gallen, and Zurich; French language - Geneva, Jura, Neuchatel, and Vaud; and Italian-language region -Ticino

^b^Self-reported age on the day of completion of the questionnaire

^c^Milk: milk and fermented milk drinks

^d^Yoghurt: yoghurt, quark, cottage cheese

^e^Cheese: soft, semisoft and hard cheeses

^f^Dairy alternatives: soya, rice and coconut-based food and beverages. Protein intake from dairy alternatives is not shown due to very low intake (< 0.5 g/1000 kcal)

^g^Numbers and percentages of participants with total protein intake below the protein recommendation DACH: < 0.8 g/kg/day for 19–65 years and < 1.0 g/kg/day for ≥ 65 years

Dairy contributed 19.3% to daily protein intake with highest contribution from cheese followed by milk and yoghurt (Table 2). Highest dairy protein intake (g/day) was in the Italian-speaking region, and lowest in French-speaking region. Data also suggest slightly higher daily as well as energy-standardized dairy protein intake in the older age groups (45–59 and 60–75 years) than younger groups (18–29 and 30–44 years).

In Table 2, the comparison of total protein intake with age-specific DACH recommendations shows insufficient protein intake in 21.3% of the population, 24.1% of women compared with 18.6% of men, and based on age groups, 40.8% of participants in age group 60–75 years compared with 13.4, 16.5 and 17.2% in age groups 18–29, 30–44, and 45–59, respectively.

**Fig. 1:**
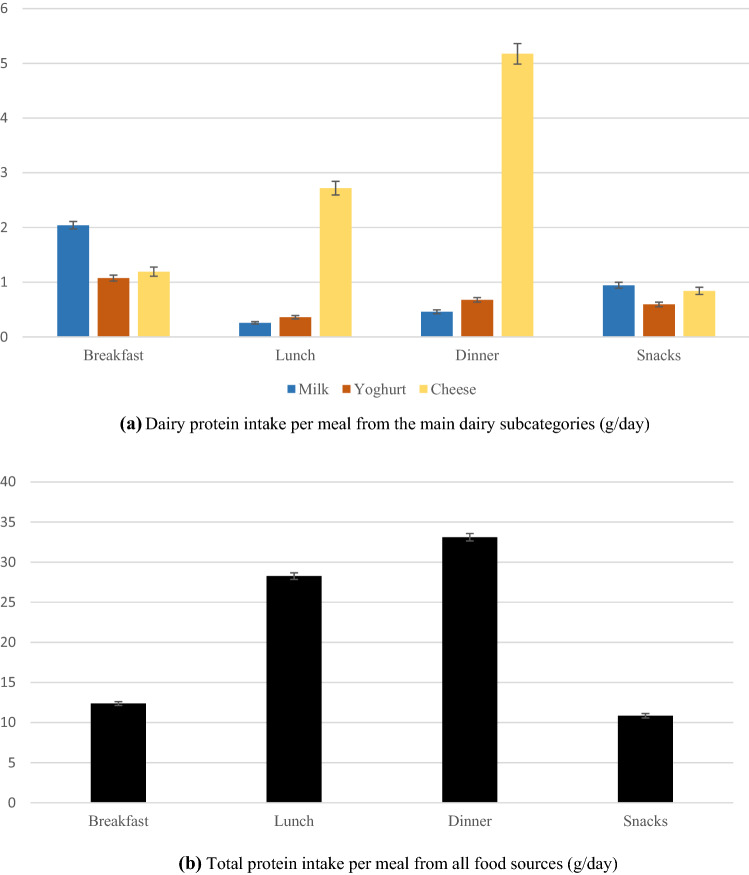
Dairy protein intake (**a**) compared with total protein intake (**b**) per meal (g/day). **a** Dairy protein intake per meal from the main dairy subcategories (g/day). **b** Total protein intake per meal from all food sources (g/day). All results are means of the two 24-HDR and weighted for sex, age, marital status, major area of Switzerland, nationality and household size, season and weekday. Error bars are standard error of the mean (SEM)

Figure 1 shows mean per-meal intake of dairy protein for each subcategory, compared with total protein intake per meal. Highest amounts of dairy protein were consumed at dinner, followed by breakfast, lunch and snacks (6.3, 4.3, 3.3 and 2.4 g/day, respectively). The protein intake from dairy subcategories varied considerably between main meals, but was similar for snacks. The greatest protein contributor was milk at breakfast (2 g/day) and cheese at lunch and dinner (2.7 and 5.2 g/day, respectively). Similar to dairy protein intake, total protein per meal was also not evenly distributed, with highest intake at dinner and lunch, and much lower intakes at breakfast and snacks (33.1, 28.3, 12.4 and 10.9 g/day, respectively).

Energy-standardized dairy protein intake showed differences between sexes and language regions (Online Resource, S3, crude data). For example, higher intake was reported for women than men at breakfast, lunch and snacks; and for the Italian region at lunch; and for the German-speaking region at breakfast compared with other regions. At all meals, data suggest similar or slightly higher energy-standardized dairy protein intake by the oldest group (60–75 years) than younger (18–29, 30–44 and 45–59 years).

Table 3 summarizes associations between energy-standardized dairy intake and sociodemographic, lifestyle and anthropometric factors. Energy-standardized dairy intake was significantly higher in women than men (20.7 g/1000 kcal [95% confidence interval (CI) 13.2; 28.1]), whereas it was lower in the French- than in the German-speaking region (− 24.3 g/1000 kcal [95% CI − 32.4; − 16.1]), in non-Swiss participants than in Swiss (− 14.0 g/1000 kcal [95% CI − 22.8; − 5.2]), for people living in a shared flat than couples without children (− 20.9 g/1000 kcal, [95% CI − 37.6; − 4.3]) and in former and current smokers than non-smokers (− 11.9 g/1000 kcal, [95% CI − 19.9; − 3.9] and − 17.2 g/1000 kcal, [95% CI − 26.5; − 7.9], respectively).

**Table 3:** Associations between energy-standardized dairy intake and sociodemographic, lifestyle and anthropometric factors

Sociodemographic and lifestyle factors	Dairy (g/1000 kcal)	
	Coefficients	95% CI
Sex		
Men	0	ref
Women	20.7	[13.2; 28.1]
Language regions^a^		
German	0	ref
French	− 24.3	[− 32.4; − 16.1]
Italian	− 7.5	[− 22.9; 7.9]
Age groups (years)^b^		
18–29	2.9	[− 9.2; 14.9]
30–44	0	ref
45–59	1.7	[− 7.6; 10.9]
60–75	3.4	[− 8.1; 14.8]
BMI categories^c^		
Underweight (BMI < 18.5 kg/m^2^)	− 12.0	[− 35.4; 11.3]
Normal (18.5 ≤ BMI < 25.0 kg/m^2^)	0	ref
Overweight (25.0 ≤ BMI < 30.0 kg/m^2^)	− 1.3	[− 9.6; 7.0]
Obese (BMI ≥ 30.0 kg/m^2^)	− 2.0	[− 13.6; 9.6]
Nationality		
Swiss only	0	ref
Swiss binational	− 9.5	[− 19.8; 0.8]
Non-Swiss	− 14.0	[− 22.8; − 5.2]
Education degree, highest degree		
Primary or no degree	− 0.5	[− 17.8; 16.7]
Secondary	0	ref
Tertiary	0.4	[− 7.3; 8.1]
Household type		
Living alone	9.2	[− 2.2; 20.6]
Adult living with parents	11.7	[− 5.0; 28.4]
Couple without children	0	ref
Couple with children	6.7	[− 2.7; 16.0]
One-parent family with children	− 1.0	[− 19.4; 17.4]
Others^d^	− 20.9	[− 37.6; − 4.3]
Gross household income (CHF/month)		
< 6000	2.9	[− 8.5; 14.4]
6000–13,000	0	ref
> 13,000	− 0.3	[− 10.9; 10.3]
Physical activity level		
Low	0	ref
Moderate	− 6.0	[− 17.4; 5.4]
High	− 1.0	[− 12.1; 10.1]
Smoking status		
Never	0	ref
Former	− 11.9	[− 19.9; − 3.9]
Current	− 17.2	[− 26.5; − 7.9]
Self-reported health status		
Very poor to medium	− 2.3	[− 13.5; 8.9]
Good to very good	0	ref
Currently on a diet		
Yes	7.3	[− 8.0; 22.5]
No	0	ref

Results of the multivariable linear regression, adjusted for all variables shown and weighted for sex, age, marital status, major area of Switzerland, household size, nationality, seasons and weekdays. Coefficients in bold are associated with a *p* value < 0.05. Missing values were replaced by multiple imputation by chained equations

*24-HDR* 24-h dietary recall, *BMI* body mass index, *CHF* Swiss Francs, *CI* confidence interval

^a^Cantons of German-language -Aargau, Basel-Land, Basel-Stadt, Bern, Lucerne, St. Gallen, and Zurich; French language—Geneva, Jura, Neuchatel, and Vaud; and Italian-language Ticino

^b^Self-reported age on day of completion of the questionnaire

^c^BMI by measured height and weight, or self-reported when measurements were not possible or pre-pregnancy weight (lactating and pregnant women)

^d^Others represents study participants living without a partner and parents (e.g. in a shared flat). Imputed values for variables with < 0.2% missing values are not shown

## Discussion

### Summary of main findings

Dairy was consumed by almost all the population in Switzerland and contributed 19.3% of daily protein, with highest protein amounts from cheese. Per meal intakes of total and dairy protein (g/day) were not evenly distributed, and revealed suboptimal total protein intakes at breakfast and snacks. Compliance to daily protein recommendations was high, except in age group 60–75 years. Significant associations were observed between energy-standardized dairy intake and sex, language region, nationality, household type and smoking status.

### Dairy consumption in Switzerland

Total dairy intake in Switzerland (216.5 g/day) was in the range of neighboring countries, similar to that in Germany (259 and 237 g/day for men and women, respectively [35]), but higher than in France (186 and 181 g/day for men and women, respectively [12]) and Italy (178 g/day [36]). Calculated on 2000 kcal, mean dairy intake in Switzerland was 199 g, which was lower than the corresponding energy-standardized mean in Denmark, but higher than that in the Czech Republic (331 g and 155 g, respectively) [37].

Comparisons of our results with other Swiss studies are limited due to the absence of previous quantitative data. However, food balance sheets showed a 4.2% decrease in per capita dairy consumption from 2010 to 2017 (248.9–238.5 kg), where the largest decrease was reported for milk (21.8%) [38]. Only 9.3% of the population met the Swiss recommendation of three daily portions, shown by consumption frequency data in the Swiss Health Study [39], and similar results were observed in the French-speaking region (8.3%, 34–74 years) [15]. Higher adherence was reported (32%) for a study group in the German-speaking region aged 50–81 years which slightly exceeded the oldest age group of our analysis [40]. Dairy intake below recommended levels was also reported in neighboring countries based on quantitative assessment methods (Germany, for women [35], France [16]) and in other countries (Spain [17], Australia [18] and the United States [19]).

An increasing trend of milk avoidance has been suggested in Switzerland, in an earlier population-based survey [41], and especially in the older population (50–81 years) [40]. In the current analysis, self-reported avoidance of dairy was stated by 16% of the population, but only 1.9% of these participants reported no dairy intake in both 24-HDR interviews (Online Resource, S2). The main reasons for avoiding dairy were intolerance (*n* = 155) and dislike of taste (*n* = 130), but also due to fat, cholesterol, allergy, and following a vegetarian/vegan diet. Although plant-based drinks have recently increased on the Swiss market [42], our analysis revealed low intake of dairy alternatives, but twofold higher in women than men. Considering their lower quantity and quality of protein, and inadequate levels of micro-nutrients compared with dairy, the nutritional consequences of replacing dairy with plant-based alternatives are a concern, especially in certain population subgroups [43].

### Associations between dairy intake and sociodemographic, lifestyle and anthropometric factors

The above discussion emphasizes the importance to gain insights on associations between sociodemographic, lifestyle and anthropometric factors and dairy intake. Energy-standardized dairy intake was significantly higher in women than in men, but daily mean intake (g/day) was slightly higher in men than women. This result could be attributed to the higher energy requirements of men than women. Higher daily dairy intake by men was also reported in national surveys in Germany [35] and France [12] based on 24-HDR, whereas the Italian survey showed higher dairy intake by women than men [36]. In these studies, data were not standardized for energy intake.

During aging, dairy may contribute to several positive outcomes, such as increased muscle mass [44] and lower risk of frailty [45]. However, digestive problems, such as lactose intolerance, may become a barrier to dairy intake [46]. Several national studies have reported reduced dairy intake in older adults, for example in Germany (65–80 years) [35], Ireland (≥ 65 years, except cheese) [47] and the United States (≥ 71 years) [19]. However, in France, dairy intake increased with age for women, whereas only cheese intake increased with age for men [12]. Our results show that dairy intake was not significantly associated with age.

Dairy products have an important role in Swiss traditional diet [48] which is reflected in our results by higher dairy intake among Swiss compared with non-Swiss citizens, and also in the German-speaking region than French- and Italian-speaking regions. Among the four main dietary patterns recently identified in Switzerland (Swiss traditional, Western 1, Western 2 and Prudent), the probability of following a Swiss-traditional diet, characterized by a high dairy intake, was also higher in the German- than French- and Italian-speaking regions [49].

Dairy intake was also significantly associated with household type and smoking status. Food intake is highly related to living conditions [50], and significantly lower dairy intake was observed in menuCH participants living in a shared flat than couples without children. Additionally, smoking status, an indicator of an unhealthy lifestyle and associated with several chronic diseases [51], was negatively associated with dairy intake. Although highly controversial, saturated fats are present in high levels in many dairy products and have been associated with cardiovascular disease [52]. Consistent with conclusions of a recent meta-analysis [53], we did not find a significant association between dairy intake and obesity or overweight.

### Protein intake from dairy, daily and per meal

Dairy provided about one-fifth of daily protein similar to results from France (21.4%) [54], but higher than Spain (16.8%) [55] and US (16.0%) [56]. While DACH protein recommendations were largely met in Switzerland, a high proportion of adults aged 60–75 had insufficient protein intake. The recently increased recommendation is based on new evidence that higher amounts of protein are needed to compensate for age-related reduction of muscle anabolic response (> 65 years) [7, 8]. Inadequate protein intake of this age group, also shown in other studies in Europe [57], could have important consequences on age-related muscle loss, sarcopenia and frailty [8, 9].

In addition to high-quality protein, a regular distribution of protein (30 g/meal) was shown to promote protein synthesis [8, 10]. Our results show that per-meal intakes of dairy protein and of total protein were not uniformly distributed across meals, with highest amounts consumed at dinner (g/day). Cheese contributed highest to dairy protein at dinner and lunch, which reflects the traditional and cultural role of cheese in Switzerland [48]. Similarly, highest amounts of dairy, especially cheese and yoghurt, were consumed at dinner in France [12], but at breakfast in Italy [11]. In both surveys, protein amounts were not reported. Milk contributed the highest amounts of protein at breakfast in Switzerland, but this may depend on breakfast type consumed, among the four breakfast patterns identified in the population [58]. Breakfast skipping was quite prevalent (35%) [58], which could lead to higher food intake at the next meal, therefore amplifying the skewed protein distribution during the day [58, 59].

Additionally, our results revealed that the suggested amounts of total protein per meal were reached at dinner and lunch, but not at breakfast and snacks. With high potential for anabolic benefits, dairy protein might be increased at meals where protein is lacking, a valuable strategy for older adults. Whereas younger adults with overall adequate protein intake might benefit by a more even redistribution of meal-protein. Overall, the above findings might be used strategically in meal planning to achieve a more evenly distributed or higher overall daily protein intake.

## Strengths and limitations

This study is based on a national, representative sample and provides a comprehensive description of dairy consumption, including energy-standardized dairy and dairy protein intake, per day and per meal based on 24-HDR. The investigation of associations between dairy intake and sociodemographic, lifestyle and anthropometric factors by multivariable linear regression was adjusted for all studied variables and weighted for representability. Among the limitations, the menuCH survey covered only 12 out of 26 cantons in Switzerland [13] and the net participation rate was at the low end (38%) of other national nutrition studies [60]. Errors due to healthy participation bias, under- or over-reporting in the 24-HDR cannot be excluded. The addition of a food frequency questionnaire, alongside the 24-HDR, would have allowed improved capture of habitual food intake.

## Conclusion

This first in-depth assessment of dairy consumption in Switzerland by sex, language region and age category is an important contribution in public health, supplying data needed to monitor dairy consumption in upcoming years. Our results show that dairy protein and total protein intake were not evenly distributed across meals. Daily protein intake was below the current recommendation in a high proportion of older adults (60–75 years). The identified sociodemographic and lifestyle characteristics of dairy intake could be used to improve nutritional recommendations, to reach population groups with low dairy and protein intake. Our study provides important supportive data to facilitate the development of meal-specific strategies aimed to optimize dairy and protein intake daily and across meals.

## Electronic supplementary material

Below is the link to the electronic supplementary material.

Supplementary file1 (DOCX 98 kb)

## Data Availability

Principles of data taransparency have been respected.

## Abbreviations

24-HDR: 24-h dietary recall
BMI: Body mass index
CHF: Swiss francs
CI: Confidence interval
SEM: Standard error of the mean

## Code availability

By request to corresponding author.
