# Supplementary material for: Daily and meal-based assessment of dairy and corresponding protein intake in Switzerland: results from the National Nutrition Survey menuCH
Source: Eur J Nutr. 2020 Oct 8;60(4):2099–109. doi: 10.1007/s00394-020-02399-7 (PMC8137467; doi:10.1007/s00394-020-02399-7)
Supplement: Supplementary file 1 — Supplementary file1 (DOCX 98 kb) [file 394_2020_2399_MOESM1_ESM.docx]

**European Journal of Nutrition**

**Daily and meal-based assessment of dairy and corresponding protein intake in Switzerland: results from the National Nutrition Survey menuCH**

**Dilara Inanir ^1^, Ivo Kaelin ^2^, Giulia Pestoni ^3^, David Faeh ^3, 4^, Nadina Mueller ^1^_,_ Sabine Rohrmann ^3^ and Janice Sych ^1^***

^1^ Institute of Food and Beverage Innovation, ZHAW School of Life Sciences and Facility Management, Einsiedlerstrasse 34, 8820 Waedenswil, Switzerland.

^2^ Institute of Applied Simulation, ZHAW School of Life Sciences and Facility Management, Schloss 1, 8820 Waedenswil, Switzerland

^3^ Division of Chronic Disease Epidemiology, Epidemiology, Biostatistics and Prevention Institute, University of Zurich, Hirschengraben 84, 8001 Zurich, Switzerland

^4^ Health Department- Nutrition and Dietetics, Bern University of Applied Sciences, Bern, Switzerland

*** Corresponding author:** janice.sych@zhaw.ch Tel.: +41 58 934 59 90

**Supplementary Material**

**
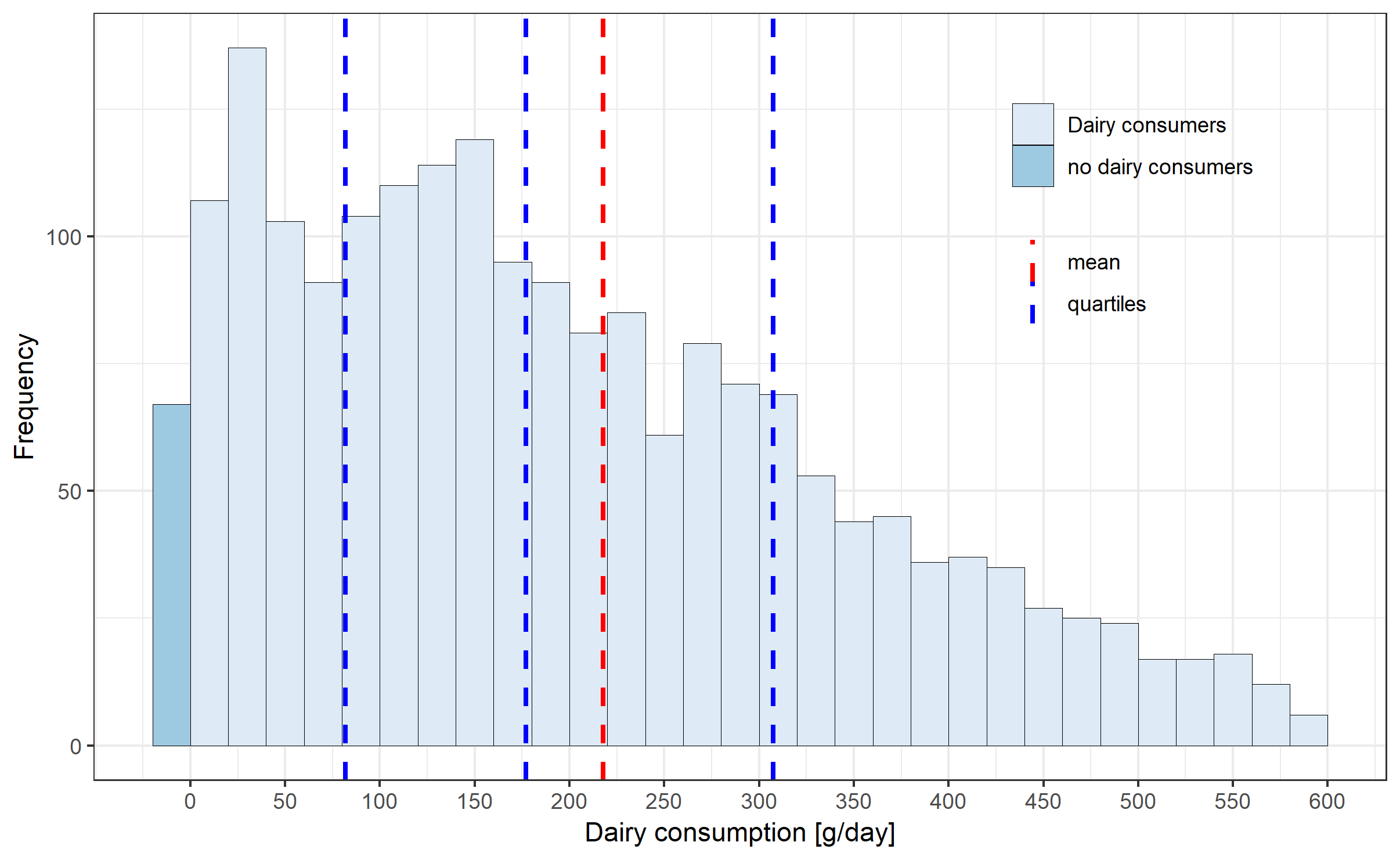
**

**S1.** Histogram of dairy intake (g/day, crude)

**S2.** Numbers of study participants who avoided dairy by 24-HDR and by self-reported statement (n and %, crude)

|  | **n** | **(%)** |
| --- | --- | --- |
| **Total^a^** | **2057** | **(100)** |
| Participants who reported dairy intake (24 HDR) | 1990 | (96.7) |
| Participant who reported no dairy intake (24 HDR) | 67 | (3.3) |
| **Total dairy avoiders^b^** | 340 | (16.5) |
| Dairy avoiders with reported dairy intake^c^ | 301 | (14.6) |
| Dairy avoiders with no reported dairy intake^c^ | 39 | (1.9) |

^a^Study participants who completed two 24-HDR. ^b^Numbers of participants who self-reported dairy avoidance for the following reasons: intolerance (n=155), not liking (n=130), fat or cholesterol (n=51), allergy (n=21), vegetarian/vegan diet (n=20), low protein intake (n=4), low salt intake (n=3) or gluten (n=2). ^c^Self-reported dairy avoiders who reported or did not report dairy intake on one or both of the interview days.

**S3.** Mean intake of energy-standardized dairy-protein at meals and snack times (Figure 3 -a, -b and -c respectively by sex, language region and age group), (g/1000 kcal, crude)

| **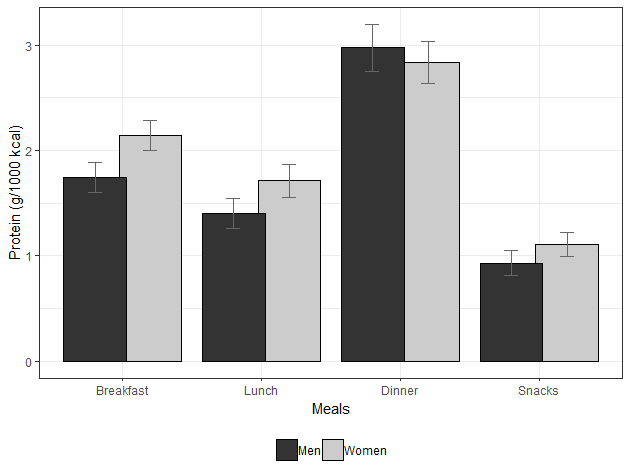a)** | **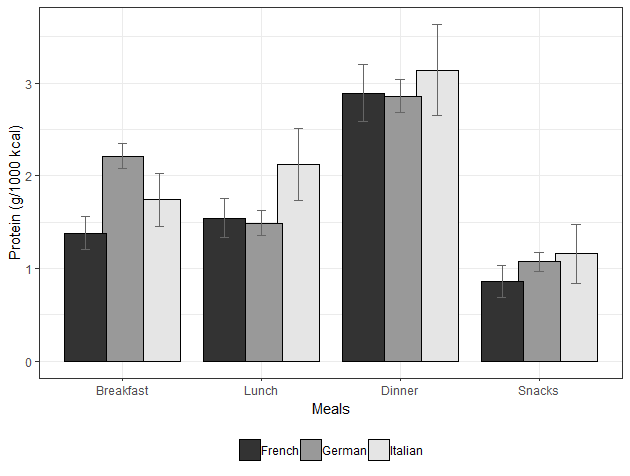b)** | **c)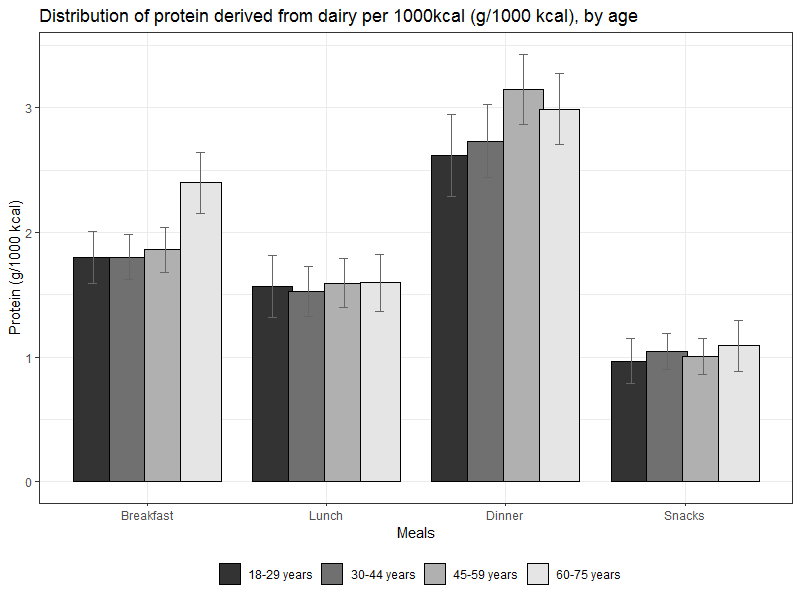** |
| --- | --- | --- |
